# Bibliometric Analysis in the Field of Rural Revitalization: Current Status, Progress, and Prospects

**DOI:** 10.3390/ijerph20010823

**Published:** 2023-01-01

**Authors:** Leng Liu, Congjie Cao, Wei Song

**Affiliations:** 1 Key Laboratory of Land Surface Pattern and Simulation, Institute of Geographic Sciences and Natural Resources Research, Chinese Academy of Sciences, Beijing 100101, China; 2 School of Economics and Management, Shihezi University, Shihezi 832003, China; 3 Department of Public Security Management, Xinjiang Police College, Urumqi 830011, China; 4 Hebei Collaborative Innovation Center for Urban-Rural Integration Development, Shijiazhuang 050061, China

**Keywords:** Bibliometrix software package, bibliometrics, rural revitalization, urban and rural development

## Abstract

Rural areas play an important role in global sustainable development. In recent years, however, rural development has experienced global crises, such as issues in public education, health care, roads, water and sanitation, along with environmental pollution and a lack of natural resources. It is therefore important to promote rural revitalization in the process of modernization. To objectively reveal the current research status in the field of rural revitalization, we analyzed relevant publications in the Web of Science from 1991 to 2021. The results are as follows: (1) In the past 30 years, the number of publications on rural vitalization has increased, and the period from 1991 to 2021 can be divided into three stages, the initial period (1991–2004), the development period (2005–2016), and the high-yield period (2017–2021). (2) Research on rural revitalization covered 60 countries or regions around the world, involving a total of 3099 authors. China, the United States, and Canada published most of the articles. (3) High-frequency keywords were migration, management, and urbanization, indicating that scientists considered the role of sustainable urban and rural development, policy formulation, and urbanization. We highlight that for the development of the field of rural vitalization, scientists need to further strengthen theoretical research, fully absorb the development achievements of advanced countries and regions, understand the laws and trends of urban and rural development in their own countries, and explore new paths to achieve rural vitalization.

## 1. Introduction

With increasing urban development globally, the gap between urban and rural areas has been intensified, and rural decline has become a common phenomenon in most countries around the world [1]. In the process of urbanization and modernization, many people move from rural areas to cities, resulting in a decline in the rural population [2,3]. This rural exodus reduces the villages’ ability to develop and threatens their sustainability and resilience, further causing environmental pollution and deep poverty [4]. In the process of urbanization and modernization, it is therefore crucial to consider and promote rural revitalization, coordinate urban–rural contradictions, and encourage balanced urban–rural development to facilitate global sustainable development [5,6].

To solve these problems, the affected countries have taken corresponding measures to promote rural revitalization and balanced urban and rural development. For example, France promoted agricultural modernization and rural development [7] by formulating policies on agricultural modernization and subsidies, and South Korea implemented the “New Village Movement” to alleviate social contradictions and promote the development of rural areas [8]. The construction of rural central villages in the UK and Japan’s “Village Comprehensive Construction Demonstration Project” aimed at the promotion of domestic rural development and achieved rural revitalization [9,10]. However, some countries/regions have experienced problems in the process of rural revitalization. For example, the formulation of a comprehensive rural development policy, namely the Rural Cooperative Investment Program (RCIP) in the United States, has been impeded [11]. Iran implemented the Rural Development Policy (RDP) to promote rural development, but after 72 years of development planning, rural Iran was still poorly developed [12].

In most parts of China, the widening gap between urban and rural areas is a common phenomenon [13,14]. Although with the implementation of China’s reform and opening up policy, rural areas have made great progress [15,16], the impacts of urbanization and industrialization on rural areas are severe [6]. In the process of China’s rapid urbanization, labor force, capital, and land flowed from rural areas to cities, whereas the cities, in turn, played a weak role in improving the conditions of the rural areas. This model was the main reason for the increasing backwardness of rural areas [17,18]. The resulting problems, such as the decline in the vitality of rural residents, the hollowing out of villages, and the increasing “village disease (A concentrated summary of social pathologies in the process of rural development)”, eventually led to rural decline and limited the sustainable development of rural areas [19,20]. Therefore, based on the experience of various countries around the world and in-depth studies of the national conditions, we can try to understand the laws and trends of urban and rural development, understand the processes and mechanisms of rural revitalization, and discuss the implementation path and direction of rural revitalization in the future.

Rural revitalization has attracted widespread attention from many organizations, research institutions, and scientists and has become the focus of geographical research in rural areas. The number of academic publications is rapidly increasing, and it is becoming increasingly difficult for researchers to track the relevant literature in their respective fields [21]. Therefore, literature reviews play an increasingly important role in synthesizing research findings from different fields to effectively use existing knowledge bases and advance a range of studies [22].

Bibliometrics is a toolkit for analyzing a range of statistical methods for scientific literature (such as scientific articles, patents, and academic proposals) [23], and has long been used as an analytical tool to systematically review literature in a given technical field or scientific discipline [24] or to address specific issues within certain frameworks of technology management [25]. Currently, this approach is receiving more attention due to the strong development of computers and the Internet [26]. Bibliometrics has been widely discussed [27,28], and one of its main advantages is that it allows the analysis of specific areas of research by considering papers, journals, authors, institutions, and countries. In recent years, the application scope of bibliometrics has been expanded, especially in the application of science and technology management and decision making, which has attracted considerable attention from relevant departments and researchers [29]. This study uses literature measurement software for the statistical analysis of the development and research status in the field of rural revitalization, facilitating the sorting, reading, and analysis of the literature in this field; generating a general picture of the field of rural revitalization research; and enabling decision makers to carry out effective quantitative management strategies backed up by scientific findings.

Research on rural revitalization is crucial in the field of sustainable development and rural geography [30,31], but there is still a lack of relevant macroscopic reviews. In addition, most studies focus on the keyword analysis, journal sources, author publications, and research countries, with few publications on the historical citation context, high-frequency keyword cluster analysis, and subject evolution. Therefore, this paper used the Bibliometrix series to analyze and visualize the literature related to rural revitalization included in the Web of Science from 1991 to 2021. The articles were comprehensively evaluated, and the possible future development direction was proposed, with the overall aim of providing a theoretical reference for the development and implementation of rural revitalization. The following questions were addressed: (1) How did the history of citations in the field of rural revitalization develop? (2) What are the main aspects of rural revitalization research? (3) What are the focus and direction of future research on rural revitalization? This study provides strategic insights into the theoretical research and development of rural vitalization and offers a reference for researchers and policy makers in the field of rural vitalization.

## 2. Data Sources and Research Methods

### 2.1. Data Sources

The Web of Science core collection is an important database for obtaining global academic information and is considered to be one of the largest bibliometric analysis databases in the world, with the most comprehensive literature types [32]. This article used the core collection in the Web of Science database as the data source; in the search term, TS was equal to “rural revitalization”. After identifying the search terms, some records were excluded, and the data were filtered. To this end, the following restrictions were implemented: (1) The retrieval time was from 1991 to 2021. Records obtained before 1991 were excluded as this year was the starting point for the analysis. The 2022 record was excluded because this year had not yet ended at the time of this study. (2) Categories that were not related to the subject of this work were excluded, such as medicine, biology, chemical science, astronomy, zoology, physics, and political science. (3) The resource type was limited to “Article” only. We only focused on the types of research articles, such as journal papers, conference proceedings papers, and review papers, excluding comments, news, letters, and other contents. After the above operations, we exported the document metadata, removed duplicates and irrelevant data, and obtained a total of 1,097 research papers in the field of rural revitalization in text format. Each document contained several elements such as the authors’ names, institution, paper title, and year of publication (Table 1).

### 2.2. Research Methods

The Bibliometrix R software package provides a set of tools for quantitative studies in scientometrics [33]. Written in the R language (an open-source environment), it is a set of integrated programs for data manipulation, calculation, and graphical display [34], which facilitates a more complete bibliometric analysis using specific tools for bibliometric and scientometrics quantitative research. Bibliometrix enables users to carry out relevant scientific measurement and visual analysis work on an interactive web interface, which reduces the user’s threshold of use and information input intensity to a certain extent [35,36]. The specific workflow of Bibliometrix mainly includes data collection, analysis, and visualization, among which data visualization is the most critical factor. Analyzing the relevant literature through the Bibliometrix software can help us to quickly understand the literature in related fields.

**Figure 1:**
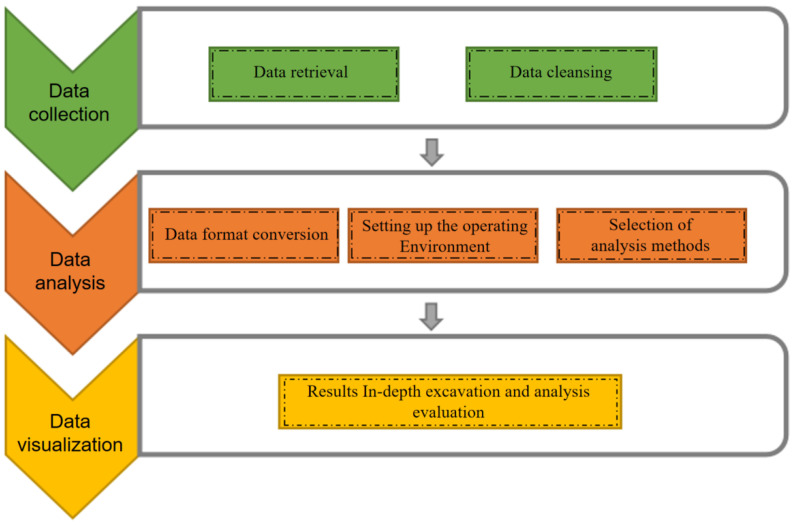
Workflow of the Bibliometrix software bibliometric analysis.

The Bibliometrix software bibliometric analysis workflow (Figure 1) includes the following: (1) Data collection (retrieval and cleansing). Many online bibliographic databases store metadata about scientific works and could be used as a source of bibliographic information, such as Clarivate Analytics Web of Science (http://www.webofknowledge.com acccessed on 22 December 2022), Google Scholar (http://scholar.google.com acccessed on 22 December 2022), and Science Direct (http://www.sciencedirect.com acccessed on 22 December 2022). With respect to data cleansing, a variety of preprocessing methods could be applied, for example, to detect duplicates and misspelled elements, including different spellings of author names, as well as forms of cited journals. (2) Data analysis. This allowed us to build the operating environment required for the software and select the analysis method according to the research content. For example, using a co-word analysis to generate a semantic map of a domain can help to understand the conceptual structure of that field. (3) Data visualization. This article extracted useful data and represented it visually or in maps, such as 2D maps, tree maps, and social networks. The data were deeply excavated, analyzed, and evaluated.

#### 2.2.1. Coupling Analysis

The concept of document coupling was first proposed by the American scholar M.M. Essler [37]. He found that the more closely related the subject or specialty of papers, the higher the amount of the same references. Two papers are coupled if the literature of the papers is at the same time (that is, the common references), and this relationship between them is referred to as “document coupling”. To put it simply, if the same references are cited in reference A and reference B, a coupling relationship is formed, and the number of the same references is called the “coupling strength”.

#### 2.2.2. Co-Citation Analysis

In 1973, Henry Small, an American intelligence scientist, proposed the concept of “Co-citation” [38]. As it is generally believed that the literature cite at the same time is more or less similar in topic, the number of co-cited publications (that is, the co-citation intensity) can measure the relevance of the literature in terms of content. Therefore, a co-citation network can be formed through the co-citation relationship among a group of publications, and the distance between nodes in the network can reflect the affinity and disaffinity of their subject content. A co-citation analysis (or co-citation cluster analysis) takes a number of publications representative of certain disciplines as analysis objects. Using multivariate statistical analysis methods, such as the cluster analysis and multidimensional scale, with the help of computers, the complex co-citation network relationship between numerous analysis objects is simplified into a small number of relationships between several groups and expressed intuitively. On this basis, the structure and characteristics of the disciplines and publications represented by the analysis objects are determined.

#### 2.2.3. H Index and G Index

The H index was proposed by the American theoretical physicist Jorge E. Hirsch in 2005 [39] and named after him. It represents the number of academic papers that have been cited at least N times, and the H index is N. Compared with the total impact factor and the total number of citations, the H index takes into account both the quantity and quality of researchers’ academic output. The G index is a concept proposed by Leo Egghe in 2006 [40]. He sorted papers in descending order according to the number of citations, and the number of citations was superimposed according to the serial number. When the cumulative number of citations is equal to the square of the serial number, the serial number value is the G index. Generally, the G index is higher than the H index.

#### 2.2.4. Multiple Correspondence Analysis

A multiple correspondence analysis (MCA) is an extension of the simple correspondence analysis to summarize and visualize tables containing more than two categorical variables. When the variables to be analyzed are categorical rather than quantitative variables, it can also be regarded as a generalization of a principal component analysis [41]. A multiple correspondence analysis (MCA) looks at multiple categorical variables and tries to find correlations between levels of these variables. The MCA extends the correspondence analysis from two variables to the case of multiple variables and can be regarded as a principal component analysis for quantitative variables. Similar to other multivariate methods, a multiple correspondence analysis is a dimensionality reduction method that represents the original multidimensional data as points in the two- or three-dimensional space.

## 3. Results

### 3.1. Annual Output Analysis of Scientific Results

**Figure 2:**
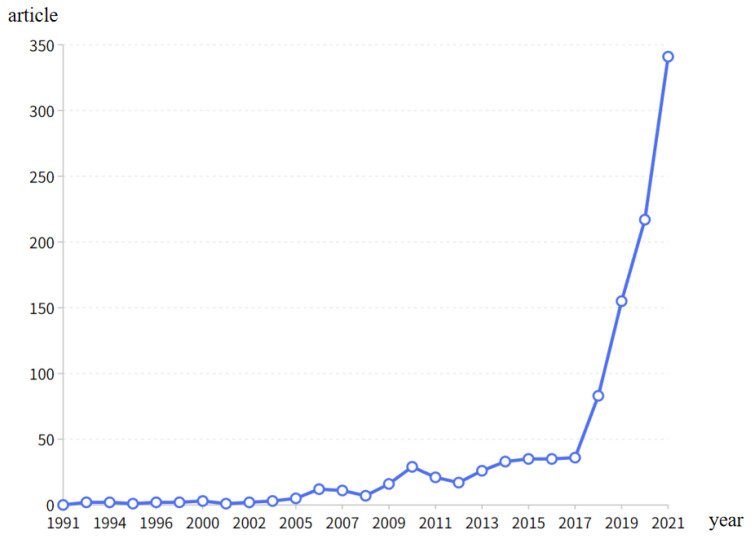
Annual scientific outputs in the field of rural revitalization from 1991 to 2021.

Based on the analysis of the annual publication volume distribution from the time series, the development trend of rural revitalization-related research can be understood (Figure 2). From 1991 to 2021, the number of publications in the field of rural revitalization fluctuated but generally showed an upward trend. In this study, the time span of the literature collection was large, and combined with the characteristics of the time series, it was divided into three research stages: 1991–2004, 2005–2016, and 2017–2021. In the first stage, only a few studies in the field of rural revitalization were published; this stage, from 1991 to 2004, can therefore be termed the “embryonic stage”. During this period, the number of publications increased slowly, and research on rural revitalization was still in its infancy, indicating that this field had not yet attracted wide attention. In the period from 2005 to 2016, the number of publications gradually increased, and the research in the field of rural revitalization entered a stage of rapid development, with an average annual publication volume of 20 articles. During the high-yield period from 2017 to 2021, the number of publications increased sharply, reaching a maximum of 341 articles in 2021. This showed that research related to rural revitalization had gradually become an important topic.

### 3.2. Citation Analysis

#### 3.2.1. Analysis of Citation Sources

**Figure 3:**
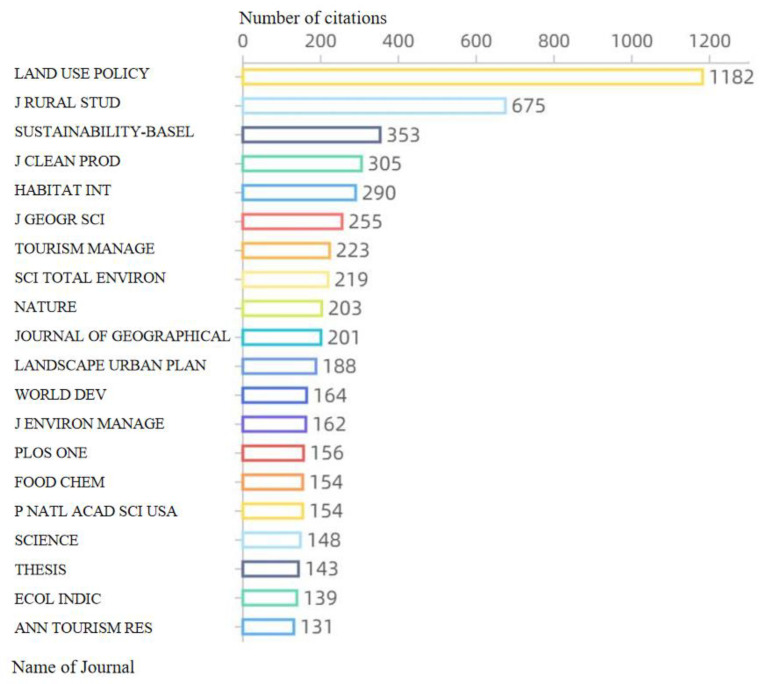
Citation sources and citations in the field of rural revitalization from 1991 to 2021. Note: The full names of the journals in the figure are as follows: *Land Use Policy*, *Journal of Rural Studies*, *Sustainability-Basel*, *Journal of Cleaner Production*, *Habitat International*, *Journal of Geographical Sciences*, *Tourism Management*, *Science of the Total Environment*, *Nature*, *Journal of Geographical Sciences*, *Landscape and Urban Planning*, *World Development*, *Journal of Environmental Management*, *PLoS One*, *Food Chemistry*, *Proceedings of the National Academy of Sciences of the United States of America*, *Science*, *Thesis*, *Ecological Indicators*, and *Annals of Tourism Research*.

Using the citation source analysis in the Bibliometrix installation package, we selected 20 nodes and found the most cited journals in the field. As seen in Figure 3, the journals *Land Use Policy*, *Journal of Rural Studies*, *Sustainability-Basel*, *Journal of Cleaner Production*, *Habitat International*, and *Journal of Geographical Sciences* were the most influential journals in this field, with *Land Use Policy* being the most cited one with 1182 citations from 1991 to 2021. The *Journal of Rural Studies*, *Sustainability-Basel*, and *Journal of Cleaner Production* were cited 675, 353, and 305 times, respectively.

#### 3.2.2. Analysis of Highly Cited Papers

Using the historical citation visualization analysis in the Bibliometrix installation package, we selected 10 nodes and found some classical studies in the field. Several classical articles appeared from 2000 to 2014 (Table 2). For example, Liu [1] published an article entitled “Introduction to land use and rural sustainability in China” in *Land Use Policy* in 2018. The author mentioned that in the context of global change and the degree of international economic integration, rural development research was the focus at the regional to global scale. In addition, he strongly called for more systematic research on land use sustainability in China and highlighted the challenges for further research on land use and rural revitalization in China. In 2014, Li et al. [35] published a paper in *Land Use Policy* titled “Community-based rural residential land consolidation and allocation can help to revitalize hollowed villages in traditional agricultural areas of China: Evidence from Dancheng County, Henan Province”. Taking Dancheng County in Henan Province as an example, this paper analyzed the current situation of rural hollowing and discussed two typical rural residential land consolidation and allocation (RRLCA) practices in China’s traditional agricultural areas (TAAs). This paper provided a comprehensive platform for promoting the construction of new rural areas, facilitating the revitalization of hollow villages, and achieving sustainable rural development. In 2016, Li et al. [42] published an article in the *Journal of Rural Studies* titled “Bottom-up initiatives and revival in the face of rural decline: Case studies from China and Sweden”. Their case studies revealed the effectiveness of bottom-up revitalization initiatives in tackling rural decline. The authors highlighted the need to not only revitalize the economy but also create an ideal rural lifestyle and develop effective rural revitalization strategies.

### 3.3. Author Analysis

**Figure 4:**
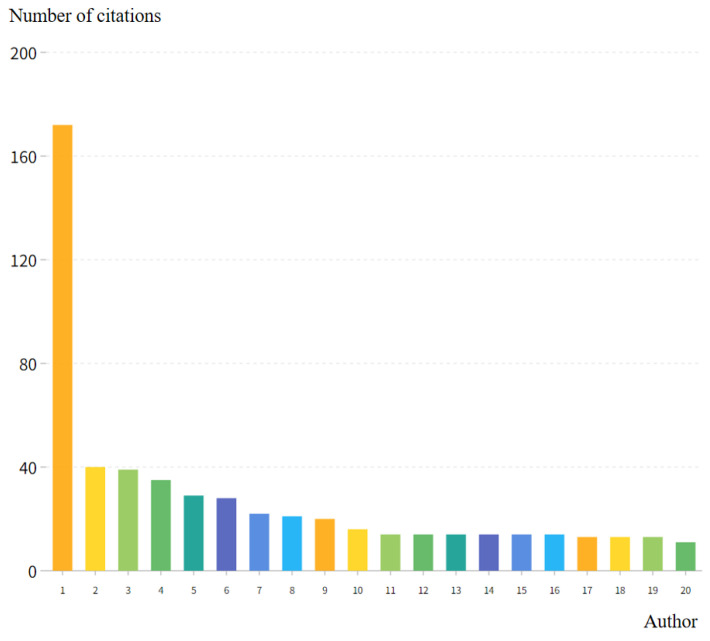
Local citation times of authors in the field of rural revitalization from 1991 to 2020 (top 10). Note: 1, Liu YS; 2, Li YR; 3, Zhou Y; 4, Li YH; 5, Long HL; 6, Westlund H; 7, Cui WG; 8, Wang YS; 9, Yang YY; 10, Zheng XY; 11, Chen L; 12, Guo YQ; 13, Lai ZX; 14, Lin YL; 15, Zhou CZ; 16, Zhu C; 17, Fu HF; 18, Li JT; 19, Li XZ; 20, Cheng MY.

A total of 3099 authors (all authors) were involved in the research papers, of which 204 authors had published articles in the field of rural revitalization. In terms of the number of published papers (Table 3), the top 10 authors published more than 5 articles from 1991 to 2021, of which Yansui Liu published 21 papers. In the field of rural revitalization, Yansui Liu ranked first in the number of publications, with an H index of 15 and a G index of 21 (the H index is a mixed quantitative indicator that can be used to evaluate the amount or level of academic output of researchers, and the G index is a derivative of the H index). Yansui Liu, Li Yr, and Zhour Y were the authors with the highest number of local citations (LCS) in this field, which were 172, 40, and 39, respectively (Figure 4).

**Figure 5:**
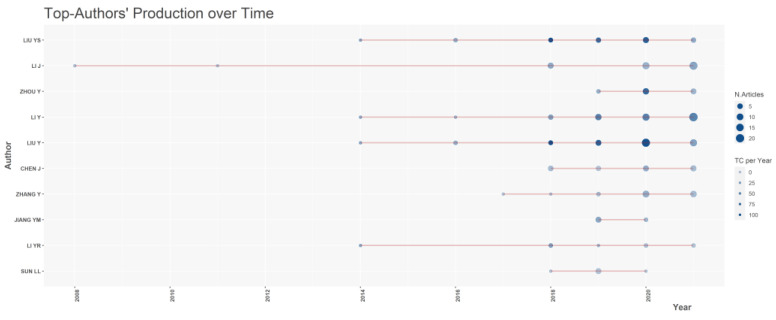
Publication trends of some authors in the field of rural revitalization from 1991 to 2021.

When considering the changes in research over time (Figure 5), scientists in the field of rural revitalization mainly published more concentrated **paper** after 2017. Among them, Liu et al. made a greater contribution during this period. In 2017, the rural revitalization strategy was officially proposed in China, and as a consequence, Liu et al. [1] published three related articles in 2018, with an average citation number of 100.4. One of these papers was “Introduction to land use and rural sustainability in China”, published in *Land Use Policy*, in which the authors mentioned the profound socio-economic transformation of China in recent decades. Rapid urbanization had severely affected rural areas, leading to increased rural problems and loss of farmland, and according to the authors, it was imperative for China to implement rural revitalization. In the same year, Yang et al. [13] published “Measure of urban-rural transformation in Beijing-Tianjin-Hebei region in the new millennium: Population-land-industry Perspective” in *Land Use Policy*, highlighting that the current urban–rural transformation had entered a critical period. The authors selected the Beijing-Tianjin-Hebei region to explore the internal mechanism of urban–rural transformation, measured the urban–rural transformation in the region, and tracked the development process of regional urban–rural changes from the essence of the urban–rural system itself. All of these papers called for more systematic research on rural areas and highlighted the challenges for further research on urban–rural relations and rural revitalization in China.

### 3.4. Analysis of Distribution Characteristics of Main Countries/Regions

**Figure 6:**
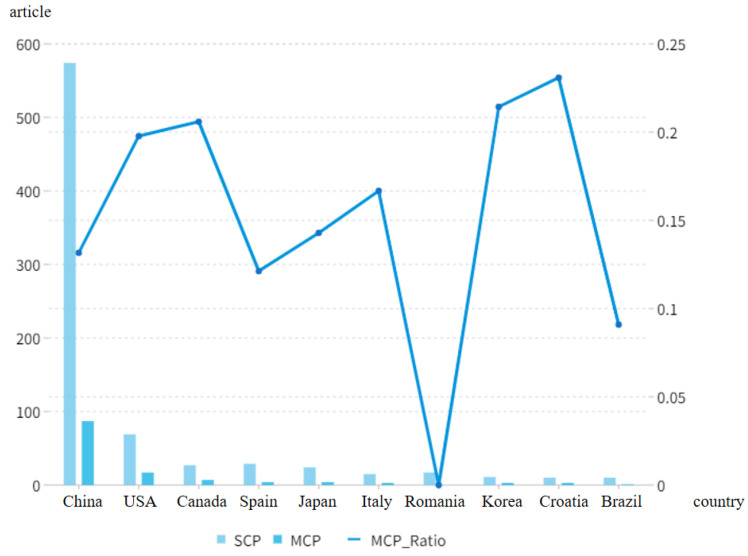
Publication cooperation of some countries in the field of rural revitalization from 1991 to 2021. Note: SCP refers to the amount of papers independently published in the country; MCP refers to the number of jointly published papers in the country; ratio indicates the percentage of joint publications in the country as a percentage of total publications.

**Figure 7:**
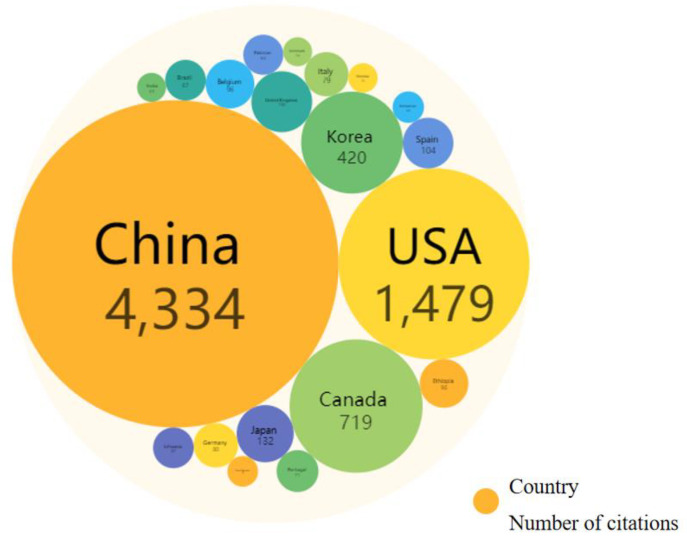
Citation times of papers in some countries in the field of rural revitalization.

The distribution characteristics of the main study countries reflected the influence of each country on research in the field of rural revitalization. The dataset used in this paper consisted of publications from 60 countries or regions, and the top 20 countries published papers as follows: 5 Asian countries (China, Japan, South Korea, India, and Indonesia); 3 American countries (United States, Canada, Brazil); 11 European countries (Spain, Italy, Romania, Croatia, Serbia, United Kingdom, Poland, Czech Republic, Germany, France, and Portugal); and 1 Oceanian country (Australia). Papers on rural revitalization were mainly published in Asia, the Americas, and Europe (Figure 6). China, the United States, and Canada published most of the papers in this field, with 661, 86, and 34 respectively. The top three cited countries were China, the United States, and Canada, with 4334, 1479, and 719 citations, respectively (Figure 7). High-yield countries also received corresponding citations, which indicated that these countries had a high influence on the field of rural revitalization. Among the above countries, Croatia, South Korea, and Canada cooperated closely with other countries, with ratio values (the proportion of joint publications of countries to total publications) of 0.23, 0.21, and 0.2, respectively. Although the total number of papers published in China far exceeded that of other countries, the ratio was 0.13, indicating that China’s research in this field was, to a large extent, not performed in cooperation. Romania had a ratio of 0, the number of independently published papers in the country was 17, and the number of joint papers was 0; given this, Romania is urged to strengthen its cooperation with other countries in the field of rural revitalization.

### 3.5. Keyword Analysis

#### 3.5.1. High-Frequency Keyword Analysis

**Figure 8:**
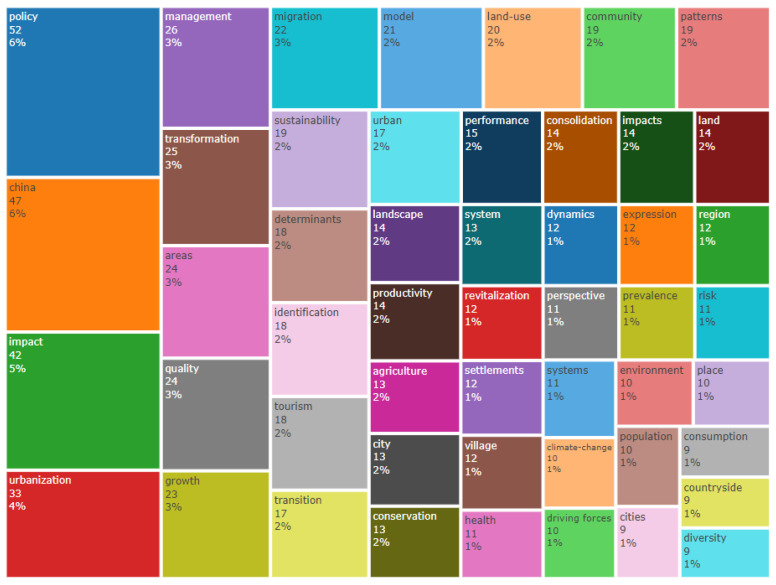
Keywords used frequently in studies on rural revitalization from 1991 to 2021.

The keywords provide a high-level summary of the content of the research paper. A high-frequency keyword analysis is an effective way to directly reflect the research hotspots of rural revitalization. In bibliometric research, publication keywords were considered to have been commonly used to reveal the structure of knowledge in the field of research [48]. The Bibliometrix software was used to analyze the high-frequency keywords and plot the word tree diagram of the top 50 keywords in the field (Figure 8). Keywords such as policy, China, impact, and urbanization appeared more frequently, 52, 47, 42, and 33 times, respectively, accounting for 21% of the total keywords. The frequent occurrence of words such as urbanization, village, transformation, and transition in the word tree indicated that rural revitalization was closely linked with the relationship between urban and rural areas, and that the promotion of integration and the integrated development of urban and rural areas were a research focus. To a certain extent, keywords such as climate change, population, and environment in the word tree indicated that these factors play an important role in rural revitalization research. In addition, sustainable urban and rural development, policy formulation, and management implementation were also frequently used.

#### 3.5.2. Cluster Analysis and Multiple Correspondence Analysis of High-Frequency Keywords

**Figure 9:**
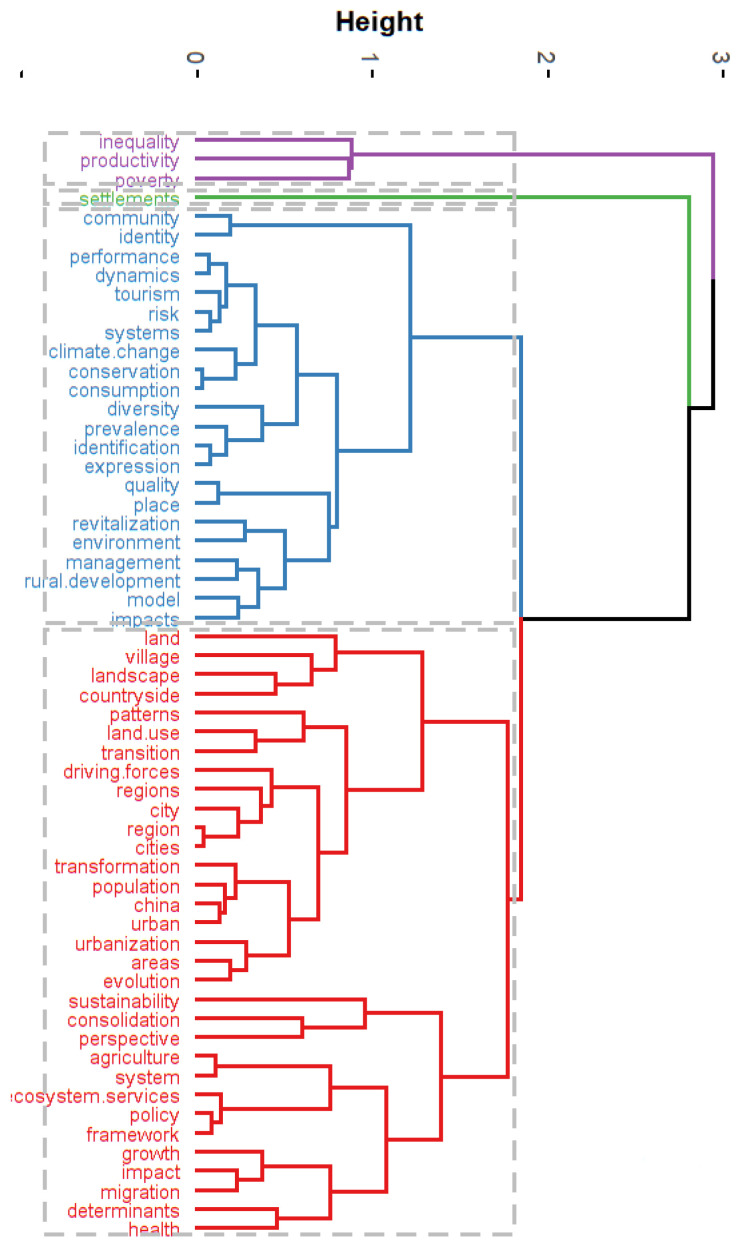
Cluster analysis of rural revitalization.

**Figure 10:**
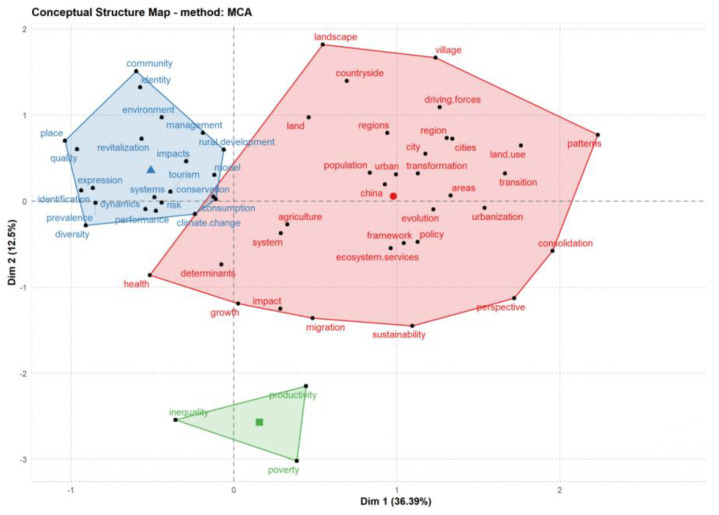
Multiple correspondence analysis of rural revitalization fields.

This study used hierarchical clustering in bibliometrics to divide keywords into several relatively small class groups according to mesh relationships, which were then merged into high-level clusters and displayed based on similarity. A cluster analysis can show the close or alienated relationship among the keywords (Figure 9). A multiple correspondence analysis (MCA) is also a commonly used method that can be employed to explore the relationships among keywords, aiming to reveal different variables or categories of the same variable in qualitative data and to reduce data dimensionality, resulting in an intuitive two-dimensional (or three-dimensional) graph that reflects similarities among keywords (Figure 10). Through this analysis, we clustered the keywords into three categories. The first category (Figure 10, red) was mainly related to policy, rural development, and sustainable development. Such research focused on releasing the vitality of rural development and promoting agricultural and rural modernization. For example, based on the “beautiful village planning strategy” against the background of the rural revitalization strategy, Zeng et al. [49], taking Shuanglongqiao Village in Xichong County, Sichuan Province as the empirical research object, summarized the planning strategies suitable for the development needs and village characteristics of Shuanglongqiao Village and put forward some references for the construction of other villages. The second category (Figure 10, blue) was mainly related to systems, models, and methods. Such studies focused on regional management and resource assessment in rural revitalization and analyzed the development potential of different regions. For example, Liu et al. [50] regarded the realization of rural revitalization as a long-term dynamic construction process, and on the basis of summary research, the authors put forward the evaluation index of rural revitalization and development potential and comprehensively evaluated the rural development level of Anhui Province from four dimensions: environmental, resource, human, and economic systems. The third category (Figure 10, blue) was mainly related to inequality and poverty in rural revitalization. Such studies evaluated the problems in the process of rural revitalization from a specific perspective and explored the underlying driving mechanisms. For example, Guo et al. [51] studied the regional differences in educational resources in the process of rural revitalization, investigated the spatial pattern of educational resources through exploratory spatial data analysis (ESDA), and explored the influencing factors of educational resources through spatial econometrics. Based on their results, measures such as village relocation and integration as well as regional development are practical ways to optimize the supply of rural educational resources and promote rural modernization, which is of great significance to rural revitalization in the new era.

## 4. Discussion

### 4.1. Research Trends in the Field of Rural Revitalization

In recent years, rural development has become the focus of global political and academic attention and discussion. Rural sustainable development and rural cultural development are now popular research fields both in China and around the world [6,52]. The rural settlement is the geographic space carrier of agricultural production, the farmer’s life, and the rural ecosystem and the core of rural man–land relationships. In recent years, research on rural revitalization has attracted widespread attention. Based on the summary of previous studies on rural revitalization, we found that in the context of the rapid advancement of industrialization, information technology, and urbanization, problems such as unbalanced urban–rural development, rural population loss, and rural aging have gradually emerged [53,54] and play a certain role in hindering rural development and policy formulation. To solve these issues, researchers have tried to evaluate the situation of rural development from different angles and to determine the weaknesses, such as those related to industrial development, ecological protection, grassroots governance, and information construction [55,56]. In recent years, research on rural vitalization has developed into a comprehensive situation of multi-perspectives and multi-disciplines, and the research perspectives have become more abundant. With more in-depth research, not only has the issue of rural sustainability been investigated, but a discussion on rural development has been initiated [57,58], covering issues such as building the rural ecotourism system [59], constructing the theoretical framework of urban–rural integration [60], and exploring the mechanism and path of rural reconstruction [61]. However, rural development is a complex and long-term process of agricultural and rural modernization and requires the continuous efforts of scientists and decision makers.

### 4.2. Comparison with Previous Studies

At present, the research perspective of rural revitalization is multi-dimensional, and numerous achievements have been made. Although many review articles have been published, most are based on a specific aspect, discipline, or research area. Zhang et al. [62] discussed the logic behind rural revitalization from the three aspects of theoretical evolution, historical evolution, and realistic development, and summarized the existing path and mode of rural revitalization. Morkunas et al. [63] conducted a systematic review of climate-smart agriculture research to determine whether agricultural revitalization can be achieved through climate-smart agriculture. Such studies enable a good understanding of the development of rural revitalization from a certain research perspective, but they ignore the systematic and integral characteristics of the research field, and it is difficult to establish a unified logical relationship between different research directions. Therefore, in our study, we sorted out and analyzed the existing literature from the perspectives of research results, researchers, research institutions, national cooperations, and future research hotspots to clarify the current development situation in the field of rural revitalization. This study therefore reflects the changes in the research content in this field from a macro-perspective, reveals the internal links among the research directions, and discusses the implementation path and future direction of rural revitalization.

### 4.3. Limitations of the Study

Although we used the core set of the Web of Science database, our study has some limitations. First, only one database was used, ignoring Google Scholar, Proquest, Springerlink, CNKI, and other databases. Second, due to the manual selection and screening of the data, the data sources are still subjective. To reduce the impact of data on this study, some work was necessary. To ensure the consistency of the study, we selected a complete time node (1991–2021) to exclude the interference of non-year-round research results. This study only included articles published before 2022. To ensure the accuracy of the study, we invited experts in the field of rural revitalization to jointly screen publication types and journal categories and manually delete duplicate and irrelevant data. To prevent data omissions, data exported from the Web of Science were saved in bulk as plain text and imported into Bibliometrix in compressed packages. In the future, on the basis of the current discussion, we will increase the selection of various databases to obtain a more comprehensive understanding of the global development of rural revitalization and promote the revitalization and development of rural areas.

### 4.4. Future Research Prospects

From 1991 to 2021, the research methods in the field of rural revitalization were gradually enriched. In the early stage of the research, a qualitative analysis was most common, while a quantitative analysis was rare. With the increasingly abundant data on rural revitalization and rural development, research on building models for empirical analysis became more apparent, and the combination of qualitative and quantitative methods was widely used [64]. Based on the analysis of the existing literature, we found that the research on rural revitalization has attracted widespread attention, for example, achieving effective governance in rural areas. In the new era, with rural revitalization and development, rural governance against the background of rural revitalization (such as inadequate rural governance mechanisms, prominent rural culture and education problems, and a lack of in-depth theoretical research on rural revitalization) needs to be studied more comprehensively [65,66]. Future research in the field of rural revitalization can be carried out from the following aspects.

(1) Strengthening the theoretical research on rural revitalization and establishing and improving the modern rural governance system. Based on previous studies, scientists have made tremendous progress in the evaluation of the development level of rural revitalization, the overall characteristics, and regional differences, but most studies are limited to the general comparison of the development levels, and research on the theoretical basis of rural revitalization needs to be further supplemented and improved [67]. The scientific output in the field of rural revitalization will continue to rise in the future, but we should not be satisfied with the number of papers only. It is crucial to consider the innovation and guiding role in the field, further improve the scientific theory of rural revitalization, and perform comprehensive research in frontier areas such as rural revitalization planning and decision making, the rural revitalization implementation path, and system modeling.
(2) With the advent of the Internet era and the rapid development of information technology, “Internet + rural revitalization” has brought numerous opportunities for agricultural and rural development [68,69]. Promoting the deep integration of next-generation information technology with agricultural production and operation and encouraging the digital and intelligent construction of rural public services and social governance are of great significance to rural revitalization. In the future, how to improve the existing functions of the Internet, contribute more efficiently to rural revitalization and development, and further strengthen the deep and effective integration of the Internet and rural revitalization are important research directions.
(3) Rural revitalization research should highlight regional characteristics and fully absorb the development achievements of advanced countries and regions. Many countries and regions have taken distinctive and successful paths in agriculture and rural development [70], and these cases should be considered in further studies and analyses, taking into account the specific characteristics of each region. The process and driving mechanisms of rural revitalization are complex. In the future, it will be necessary to further explore the driving mechanisms of different regions and the different factors. Therefore, in promoting rural construction, we should proceed from reality and innovate the regional rural development mode according to the different rural geographical locations and agricultural resources.
(4) We should strengthen cross-field and cross-regional research on rural revitalization. Rural revitalization should be an integral part of the global governance system, and it is urgent to actively integrate it into the world’s rural development system and promote international cooperation and innovative development in an orderly manner. At present, although domestic scientists have achieved considerable research efforts, an academic interaction is lacking. However, such an interaction not only promotes an improvement of the original research methods and conclusions, but also explores new problems and can offer new ideas. Therefore, for scientific, profound, and diversified research on rural vitalization strategies, an academic exchange among scholars is essential. It is necessary to strengthen cooperation among experts and scholars from different disciplines, fields, and countries; promptly incorporate innovative methods and ideas from other disciplines; focus on cross-disciplinary research; and promote in-depth research on rural revitalization strategies. This is of great significance for sharing research resources and promoting sustainable rural development.

## 5. Conclusions

In this study, based on the literature related to rural vitalization in the Web of Science database and combined with the advantages of different bibliometrics software packages, we quantitatively analyzed the research status in the field of rural vitalization. From 1991 to 2021, the number of publications in this field showed an overall upward trend. The development can be divided into three stages: the low-yield period (1991–2004), the development period (2005–2016), and the high-yield period (2017–2021). The citation frequency increased over time, which was partly closely related to the development of this research field. Regarding the citation sources, the journals *Land Use Policy*, *Journal of Rural Studies*, *Sustainability-Basel*, and *Journal of Cleaner Production* largely influenced the development of this research field, with 1,182, 675, 353, and 305 citations, respectively. A total of 3,099 authors were involved in the respective publications, with an H index of 15 and a G index of 21. Papers published in the field of rural revitalization from 1991 to 2021 came from 60 countries or regions, mainly from Asia, the Americas, and Europe. China, the United States, and Canada published the most papers in this field, with 661, 86, and 34 publications, respectively. Romania’s ratio was 0, indicating that this country needs to strengthen its cooperation with other countries in this research field.

In the initial stage, the research was mostly exploratory, without a mature framework and concept. Over time, scientists gradually deepened their research, and currently, the scientific output in the field of rural revitalization is on the rise. However, in the future, scholars should not only focus on the increase in the number of publications but are urged to pay more attention to the quality of papers and their innovative and guiding role in this field. Rural revitalization is closely linked to urban and rural areas, and how to promote the integration and integrated development of urban and rural areas will be the focus of future research. Based on the quantitative analysis of the research status in the field of rural revitalization, we urge scientists to strengthen cross-field and cross-regional research, focusing on the important role of the factors affecting sustainable urban and rural development, and on the policy making and management implementation in the field of rural revitalization.

## Figures and Tables

**Table 1:** Data document identifier and meaning.

Serial Number	Content	Serial Number	Content
FN	Clarivate Analytics Web of Science	TC	0
VR	1	Z9	0
PT	C	U1	11
AU	Zhou, F	U2	30
AF	Zhou, Fang	PU	FRANCIS ACAD PRESS
BE	Zhu, Z	PI	LONDON
TI	Strategy of Rural Leisure Tourism Promotes the Implementation of Rural Revitalization	PA	35 IVOR PL, LOWER GROUND, LONDON, NW1 6EA, ENGLAND
GA	BM1IP	BN	978-1-912407-23-1
SO	2018 INTERNATIONAL WORKSHOP ON ADVANCES IN SOCIAL SCIENCES (IWASS 2018)	PY	2019
LA	English	BP	880
DT	Proceedings Paper	EP	884
CT	International Workshop on Advances in Social Sciences (IWASS)	DI	10.25236/iwass.2018.187
CY	DEC 12–13, 2018	PG	5
CL	Hongkong, HONG KONG	WC	Social Sciences, Interdisciplinary
DE	Leisure Tourism; Implementation Method; Rural Revitalization	DA	17 August 2022
UT	WOS:000459861700187	SC	Social Sciences-Other Topics
RP	Zhou, F, Xian Peihua Univ, Xian 710000, Shaanxi, Peoples R China.	EF	
WE	Conference Proceedings Citation Index-Social Science & Humanities (CPCI-SSH)	ER	

Note: The table takes the *Strategy of Rural Leisure Tourism promoting the Implementation of Rural revitalization* as an example. FN: file name; VR: version number; PT: publication type; AU, author; AF, full name of author; BE, editor; TI, literature title; SO, publication name; LA, language; DT, literature type; CT, conference title; CY, date of meeting; CL, conference venue; DE, author’s key words; RP, corresponding author’s address; TC Web of Science, citation frequency count of core set; Z9, total cited frequency; U1, use times (last 180 days); U2, times of use (2013–present); PU, publisher; PI, the city where the publisher is located; PA, address of publisher; BN, International Standard Book Number (ISBN); PD, date of publication; PY, year of publication; VL, volume; IS, period; BP, start page; EP, end page; DI, digital object identifier (DOI); PG, number of pages; WC, Web of Science category; SC, research direction; GA, document delivery number; UT, stash number; ER, end of record; EF, the file is complete.

**Table 2:** Highly cited articles in the field of rural revitalization from 1991 to 2021 (Top 10). LCS = number of local citations; GCS = global citation score.

DOI	Year	LCS	GCS	Source	Reference
10.1016/j.landusepol.2018.01.032	2018	49	346	*Land Use Policy*	[1]
10.1016/j.landusepol.2014.02.016	2014	22	213	*Land Use Policy*	[35]
10.1016/j.jrurstud.2016.07.004	2016	16	97	*Journal of Rural Studies*	[42]
10.1007/s11442-019-1619-9	2019	13	51	*Journal of Geographical Sciences*	[43]
10.1016/j.jrurstud.2019.03.003	2019	12	151	*Journal of Rural Studies*	[44]
10.1016/j.tourman.2017.04.003	2017	11	142	*Tourism Management*	[45]
10.1016/j.landusepol.2018.08.005	2018	11	74	*Land Use Policy*	[13]
10.1016/j.landusepol.2018.12.013	2019	11	53	*Land Use Policy*	[46]
10.1016/j.landusepol.2019.104330	2020	11	88	*Land Use Policy*	[47]
10.1016/j.landusepol.2019.104379	2020	9	72	*Land Use Policy*	[15]

**Table 3:** Top 10 authors that published articles in the field of rural revitalization from 1991 to 2021 (top 10).

Author	Articles	Author	Articles
Liu YS	21	Chen J	9
Li J	13	Zhang Y	9
Zhou Y	12	Jiang YM	8
Li Y	11	Li YR	8
Liu Y	10	Sun LL	8

## Data Availability

All relevant datasets in this study are described in the manuscript.
